# Women’s descriptions of childbirth trauma relating to care provider actions and interactions

**DOI:** 10.1186/s12884-016-1197-0

**Published:** 2017-01-10

**Authors:** Rachel Reed, Rachael Sharman, Christian Inglis

**Affiliations:** 1 University of the Sunshine Coast, 90 Sippy Downs Drive, Sippy Downs, QLD 4556 Australia; 2 The University of Notre Dame, 160 Oxford St, Sydney, NSW 2010 Australia

**Keywords:** Childbirth, Trauma, Maternity care

## Abstract

**Background:**

Many women experience psychological trauma during birth. A traumatic birth can impact on postnatal mental health and family relationships. It is important to understand how interpersonal factors influence women’s experience of trauma in order to inform the development of care that promotes optimal psychosocial outcomes.

**Methods:**

As part of a large mixed methods study, 748 women completed an online survey and answered the question ‘describe the birth trauma experience, and what you found traumatising’. Data relating to care provider actions and interactions were analysed using a six-phase inductive thematic analysis process.

**Results:**

Four themes were identified in the data: ‘prioritising the care provider’s agenda’; ‘disregarding embodied knowledge’; ‘lies and threats’; and ‘violation’. Women felt that care providers prioritised their own agendas over the needs of the woman. This could result in unnecessary intervention as care providers attempted to alter the birth process to meet their own preferences. In some cases, women became learning resources for hospital staff to observe or practice on. Women’s own embodied knowledge about labour progress and fetal wellbeing was disregarded in favour of care provider’s clinical assessments. Care providers used lies and threats to coerce women into complying with procedures. In particular, these lies and threats related to the wellbeing of the baby. Women also described actions that were abusive and violent. For some women these actions triggered memories of sexual assault.

**Conclusion:**

Care provider actions and interactions can influence women’s experience of trauma during birth. It is necessary to address interpersonal birth trauma on both a macro and micro level. Maternity service development and provision needs to be underpinned by a paradigm and framework that prioritises both the physical and emotional needs of women. Care providers require training and support to minimise interpersonal birth trauma.

## Background

Around one third of women experience trauma whilst giving birth [1, 2]. A traumatic birth experience is associated with postpartum mental health problems, including depression and post traumatic stress disorder [PTSD] [1, 3–6]. Poor mental health in the postnatal period can alter a woman’s sense of self, and disrupt family relationships [7–10]. Difficulties with early mother-baby bonding can negatively influence a child’s social, emotional and mental development [11]. In addition, the experience of a traumatic birth can influence a woman’s future decisions regarding where, how, and with whom she gives birth [12, 13]. For example, women may choose to birth at home to avoid repeating a traumatic hospital experience [14]. Jackson et al. [15] found that the decision to freebirth (give birth without a professional care provider) can be influenced by previous birth traumatic. Therefore, the consequences of a traumatic birth experience can be substantial and wide-ranging for women and their families.

Birth trauma has been associated with medical intervention and type of birth [5, 16, 17]. It has been defined as a perception of ‘actual or threatened injury or death to the mother or her baby’ [18]. However, Beck [19] argues that the perception of trauma is in the ‘eye of the beholder’, and should be defined by the woman experiencing it. Qualitative studies exploring women’s experiences of traumatic birth identify interactions with care providers as a more important factor than medical intervention or type of birth [20–23]. For example, a perceived lack of control and involvement in decision-making can contribute to the experience of trauma [21, 23]. A study by Thomson and Downe [20] found that trauma was related to ‘fractured interpersonal relationships with caregivers’, and that women felt disconnected, helpless and isolated during birth. Whilst not all traumatic birth experiences result in PTSD, two quantitative meta-analyses identified that negative care provider interactions are a significant risk factor for PTSD [5, 17]. A study by Harris and Ayers [24] also found that the strongest predictor of developing birth related PTSD was interpersonal difficulties with care providers, in particular experiencing a lack of support.

A recent Cochrane Review [25] concluded that women require improved emotional support during birth from their care providers to reduce the risk trauma. Health care professionals have an ethical, legal and professional obligation to provide safe and respectful care [26–28]. In order to improve care, it is important to understand what interactions and actions are associated with trauma [20]. This paper focuses on traumatic care provider actions and interactions from the perspective of the women experiencing them. The findings contribute to the body of literature examining women’s experiences of traumatic birth; and to an understanding of how care providers influence women’s perceptions of trauma. This paper presents a subset of findings from a large mixed methods study that investigated parental mental health following traumatic birth. The quantitative findings have not yet been published. The qualitative findings concerning paternal mental health are reported elsewhere [29]. This paper presents the qualitative findings relating to women’s descriptions of birth trauma involving care provider actions and interactions.

## Methods

The mixed methods study involved parents completing an online survey, and additional face-to-face interviews with fathers [29]. The online survey included questions on demographics, descriptive birth assessments, parent-infant attachment, partner relationship quality, current mental health, and coping strategies used after the trauma. In addition, the survey incorporated a question about the experience of birth trauma with space for a written response. A qualitative approach was taken to explore women’s written descriptions of trauma. The area of interest in this aspect of the study was women’s experiences of trauma, rather than outcomes associated with trauma. The majority of qualitative data related to care provider actions and interactions, and this paper presents themes relating to this data.

### Participant recruitment

Participants were recruited via online social media forums such as Facebook, Twitter and a midwife’s blog site. Inclusion criteria was that participants were over 18 and had experienced a traumatic birth. A definition of a traumatic birth was not provided in order to capture what participants themselves considered’trauma’ [19]. There was no exclusion criterion for time since the birth, as women’s memories of childbirth remain strong over time [30]. Participant information detailing the research question and aims was provided on the first page of the online survey. In order to obtain consent, participants were required to read an online consent form and ‘click’ agree prior to accessing the survey.

### Data collection

After consenting to participate, participants completed an online survey administered through the program Survey Monkey. The survey included demographics (eg. age, relationship status) and information such as type of birth (eg. caesarean, vaginal); place of birth (eg. public hospital, home); and admission of baby to special care (Table 1). The quantitative element of the study comprised of a number of psychological assessment tools: Maternal Postnatal Attachment [31]; Quality of Marriage Index [32]; Depression Anxiety Stress Scale-21 [33]; Posttraumatic Stress Disorder Checklist-5 [34]; and The Brief Cope index [35]. The qualitative element of the study involved women responding in their own words to the question ‘describe the birth trauma experience, and what you found traumatising’. The mean length of written responses was 69 words.

**Table 1:** Demographics and type of birth

Age	
Range = 18 to 77 years (*Mean* = 33.13)	
	N (%)
Marital status	
Married	798 (77.5%)
De Facto	124 (12.5%)
Single	42 (4.1%)
Divorced	21 (2%)
Separated	23 (2.2%)
In a relationship but not living together	11 (1.2%)
Widowed	2 (0.2%)
Engaged	4 (0.3%)
Number of children	
0	7 (0.7%)
1	409 (39.4%)
2	345 (34%)
3	159 (15.3%)
4	70 (6.8%)
5	23 (2.2%)
> 5	17 (1.5%)
Region of origin	
Australia and Oceania	386 (36.8%)
North America	347 (34.2%)
Europe	253 (25.5%)
South America	23 (2.1%)
Asia	8 (0.9%)
South Africa	7 (0.5%)
Middle East	2 (0.2%)
Education	
Did not finish high school	14 (1.4%)
Finished high school	196 (19.1%)
Trade or technical qualification	157 (14.1%)
Undergraduate degree	395 (39.1%)
Postgraduate degree	264 (26.4%)
Type of birth	
Unassisted vaginal birth	271 (34.3%)
Assisted vaginal birth (ventouse or forceps)	176 (22.4%)
Planned caesarean	47 (6%)
Unplanned caesarean	290 (37%)
Place of birth / transfer	
Public hospital	542 (69%)
Private hospital	115 (14.6%)
Birth centre	12 (1.5%)
Planned birth centre transfer to hospital	23 (2.9%)
Homebirth	29 (3.7%)
Planned homebirth transfer to hospital	63 (8%)
Unplanned out of hospital birth	1 (0.1%)
Admission of baby to special care nursery	
Yes	269 (34.4%)
No	512 (65.6%)

### Data analysis

Women’s descriptions of trauma were analysed using a six-phase inductive thematic analysis process described by Braun and Clarke [36]. Phase one involved becoming familiar with the data by reading and re-reading; and noting initial ideas. In phase two initial codes were generated and data relevant to each code was collated. Phase three of the process involved collating the codes into potential themes. These themes were reviewed in phase four to ensure they were consistent in the coded extracts and across the entire data set. In phase five themes were defined and named using words and phrases. Phase six involved selecting extract examples to illustrate the themes, and relating the analysis to the research question and the literature. Three researchers participated in the thematic analysis process to ensure consistency in analysis and findings.

### Findings

A total of 943 women completed the online survey from around the world. The majority of participants were from Australia and Oceania (36.8%), North America (34.2%) and Europe (25.5%). A small number of participants were from South America (2.1%), Asia (0.9%), South Africa (0.5%) and the Middle East (0.2%) (Table 1). The majority of participants gave birth in a public hospital (69%) and either had an unplanned caesarean (37%), or an unassisted vaginal birth (34.3%) (Table 1). In addition, 34.4% of participants reported that their baby was admitted to special care nursery.

Of the 943 participants, 748 (79%) responded to the qualitative question ‘describe the birth trauma and what you found traumatising’. A third of respondents described events such as premature labour, haemorrhage or concerns regarding their baby’s wellbeing. However, the majority (66.7%) described care provider actions and interactions as the traumatic element in their experience. From the data relating to interpersonal factors, four overarching themes were identified from the descriptions. The themes are presented below with illustrative data using the participants’ own words, therefore spelling and grammar varies. The term ‘care provider’ is used to refer to the professional responsible for the woman’s care. In the women’s accounts care providers included obstetricians, midwives and nurses.

### Prioritising the care provider’s agenda

Women described how care providers prioritised their own agenda over the needs of the woman. In some cases it was made clear to women that their labour was keeping the care provider from something, or someplace they would rather be:

I found my OB’s lip service to my wishes and then his switch against them traumatic. I found the comment “let’s get this over and done with, I have a golf game to get to” traumatic… (045)

… after an OB coming in and telling me that she would like me to deliver by 5 pm because she wanted to go home, I just burst in to tears… (549)

Women felt that they were subjected to unnecessary and unwanted medical interventions in order to meet the needs of their care providers:

I begged not to have a c section, neither I nor my baby were in distress or danger, but because the doctor was ready to go home, he did a terrible section that resulted in almost a year of recovery. (220)

I was steamrolled with unnecessary intervention and didn’t get to speak with a doctor about my options, risks vs benefits… I feel like the nurses, doctors and hospital only did what was in their best interest, not mine… It was a nightmare. (381)

Some women described how they became a learning resource for the benefit of hospital staff. For example, care providers offered other staff the opportunity to practice without seeking women’s permission:

… the doctor asked a student nurse, first day on the job, if she wanted to suture my episiotomy incision. (644)

… 20 people in theatre and half were sitting down on phones and chatting away while I had someone training with forceps on me… (867)

One woman described feeling like she “… was part of an experiment” (565) rather than a woman giving birth. In particular, women experiencing unusual births became a spectacle for others to watch:

… I was a looking point for students and anyone who hoped to witness a twin vaginal birth and a breech birth. (523)

One woman wrote about how the room filled with staff hoping to watch her give birth to her breech baby:

… and the amount of people that filled the room to watch a vaginal breech delivery, when I failed at this, everyone left. (662)

When she was unable to provide this learning opportunity she no longer warranted being an object of observation. The value of her birth experience for others appeared to be based on what she could provide in terms of a learning experience.

### Disregarding embodied knowledge

Many of the descriptions involved women’s own embodied knowledge being disregarded in favour of their care provider’s assessment of events:

… I felt like I was being told I was silly for thinking I was in labour and that this awful pain was nothing to be worried about. My opinion was dismissed and ignored as I was just a first timer… (436)

In particular ‘being in labour’ was a contested area. Women’s perceptions of being in labour were based on their embodied experience, whereas care provider’s perceptions were based on clinical findings. For example, one woman was considered to ‘not be in labour’ because her cervix was not dilating according to care provider’s expectations:

Hospital staff did not listen to me, didn’t trust me to know my body. Dismissed me as a first time mother who was over reacting. In actual fact I dilated from 0 to 6 in just over an hour. The hospital midwives told me that I was just feeling the period pain associated with early labour and induction… (485)

Another woman described how her midwife determined she was not contracting, therefore not in labour, based on an abdominal palpation:

Was going into premature labour and midwife palpated during a contraction and stated I was not having them. Eventually went into labour as they ignored me… Although not traumatic in medical terms, felt completely disgruntled that my journey was not taken on own merits and was completely ignored as a woman during labour. (061)

Both of these women considered themselves to be in labour, and having their embodied knowledge disregarded was traumatic.

Embodied knowledge was also dismissed when women experienced an urge to push before care providers considered it appropriate. Women were instructed to ignore what was happening in their body and stop pushing:

Told to stop pushing and… being told what to do when my body was telling me differently. (248)

Being told to stop pushing when baby was clearly on its way. Being told I had a long way to go when baby was on the way out. (436)

Care providers used clinical assessments (vaginal examinations) to determine whether pushing was appropriate. Based on the findings of these clinical assessments women were ordered to over-ride their own bodily urges:

… I had the strongest urge to push, the midwife on staff insisted on an internal examination to check dilation, she told me if I pushed now I would end up with an emergency caesarean due to my cervix swelling. She then spent the next hour yelling at me not to push and trying to talk me into an epidural (I was trying my hardest to not push but my body kept taking over). I was begging to be allowed to push…. (932)

In some cases women described feeling that the wellbeing of their baby was in danger. When they attempted to alert care providers their embodied knowledge was disregarded:

… I felt like everything was going wrong and found that distressing. I felt like people didn’t believe me when I said something didn’t feel right. (851)

… My baby was in distress and had mec liquor and in all honesty probably should’ve been sectioned, at this stage I was begging for one as I knew something was wrong with my baby but they refused… (732)

In these descriptions women’s own assessment of labour progress and fetal wellbeing was not valued or acted on which caused trauma.

### Lies and threats

Women perceived that they were being lied to by care providers to coerce them into agreeing to unnecessary interventions:

It was not the birth itself that I found traumatic, rather the way we were treated by the midwife. Being lied to in order to speed up my labour unnecessarily and putting me and my baby at risk. (015)

All of this is avoidable and unnecessary, if only we had known… I was forced into interventions that I believed were unnecessary. I was also lied to many times by the doctors. (857)

They also described how care providers threatened them in order to coerce them into undergoing procedures:

My daughter was breech… I was told that if I didn’t consent to the cesarean before labor started then they would perform a cesarean without my consent under general anesthesia when I arrived (267).

In this case, the woman was threatened with surgery against her wishes. Other women were threatened with having their baby taken from them if they did not comply with proposed interventions:

Psychological coercion - ie “if you do not consent to syntocin OR a c-section then we can get our friend the psych registrar down here to section you - then we can do whatever we want to you but you may not be able to keep your baby” - All I wanted was to let my body go into labour naturally - my baby was not in distress… (186)

I was bullied into an induction late on a Sunday night and then told I would be kept over night. I wasn’t aware when I finally agreed to be induced after quite some time of being threatened with DoCS [Department of Child Safety] etc. (400)

The most common threats described by women related to the wellbeing of the baby. Some women used the term ‘dead baby threat’ to describe how they were coerced, for example: “dead baby threats to gain consent…” (860); and “forced into c section with dead baby threat…” (223). Some care providers asked women if they wanted their baby to die when they declined an intervention:

…Being bullied into interventions with such wording the following: “Do you want a dead baby?”… (919)

Women felt that care providers were lying about the risks to the baby in order to pressure them into complying. They did not believe their babies were in danger, and in some cases had evidence that their care provider’s assessment was incorrect:

…I was basically told that if I didn’t have a c-section on their timetable I would kill my baby, even though they couldn’t tell me what exactly was “wrong” as to why I was not delivering vaginally… They broke me down gradually until they declared my baby was “in distress” (she wasn’t… I could see the screens). (559)

… Lots of coercion and being told my baby would die if I didn’t consent to the c-section. She was born with apgars of 9 and 9. (194)

Being lied to and threatened contributed to the experience of trauma, particularly when it involved the wellbeing of the baby.

### Violation

Many women described their birth experience as ‘violating’. A lack of control appeared to be associated with a sense of violation. For example, one woman described that she felt “…out of control and violated” (660). In these descriptions, care providers carried out actions against the explicit wishes of the woman:

…All in all, I felt very bullied, and even violated… It was the feeling of disempowerment and not having the right to do with my body what I wished - and that someone else could force me to do something against my will. (731)

I felt violated, and angry that I should have to defend myself and my body while I was trying to push my baby out. (733)

The descriptions of what care providers did to women were, in many cases, graphic and violent. For example, one woman wrote “…couldn’t be tubed nurses manually choked me out” (490). Another wrote that she was “… assaulted vaginally by medical staff during crowning” (295). These descriptions focused on the manner in which the care provider acted, in addition to their actions:

… She was very rude and condescending, both to myself and to my midwife. She proceeded to dig out my uterus without any numbing medication. It was horrifying… (431)

…The pain was not the traumatic bit, it was the way that I was treated during my labour. I was 20 years old. I had more midwives than I can count, attempt an internal examination and one yelled at me to ‘relax!’ because she couldn’t force her fingers in. She was a bloody bitch to put it lightly. (256)

One woman described how her obstetrician assaulted her to gain her compliance to induce labour:

She said she wanted to do one more cervical check. I consented and when she did it, she grabbed my cervix and pinched it. She would not let go until I consented to letting her break my water. I was in tears from the pain, screaming, begging and sobbing for her to let go and get her hand out of my vagina. She would not let go until I consented, which I finally did. (997)

A number of women described how they screamed ‘no’ as care providers carried out procedures. For example, one woman told her care provider “expressively” that she “didn’t want any vaginal examinations” (413). Her care provider persuaded her to have a vaginal examination telling her that they “would be very gentle and would stop if it was too much”. However her wishes were not respected during the examination:

I was crying and screaming in pain telling her no and to stop and she carried on, my husband shouted at her to leave me alone and she carried on. (413)

Another woman described how her doctor failed to respond to direct requests, and then to screams for her to stop:

The doctor would not get her fingers out of my vagina even when directly told. After it was discovered that I suffered tearing, I wanted the tearing to be healed on its own - no stitches, but she and another doctor stitched anyway, despite my screaming at them to stop. (445)

In addition, some women wrote about being ‘held down’ while care providers carried out procedures against their will:

…Being pinned down by 4 midwives (forcing an unnecessary oxygen mask on me just so my screams of ‘no’ were muffled) and my husband so the consultant could examine me against my will. (888)

…At one point, 3 nurses physically held me down despite my protests that I couldn't breathe and needed a minute to catch my breath before the procedure (AROM). They held me down until the doctor was finished… (491)

Women described how equipment tethered or tied them to the bed during labour: “was tethered to the bed during an induction…” (328), and “I was tied to the bed, forced to lay on my back…” (418). Women experienced being forced into birth positions: “screaming, lots of people, nurses forcing me down and ripping my legs open…” (565). In particular, care providers made women lie on their backs:

During birth, multiple nurses screamed in my face “PUSH!!!” and flipped me onto my back and forced my legs open, holding me down… (414)

In describing their experiences women used words such as “humiliating” (561); “belittled” (520); “brutal and barbaric” (132). Some described “being treated like a piece of meat” (979), or an animal:

…I was treated like a cow having trouble calving, and felt abused and humiliated. (222)

A number of women used language associated with sexual assault and rape, writing that they felt: “…raped and mutilated” (376), “… violated and damaged” (119), “…violated and scared and disgusting” (423). Women who had previously experienced sexual abuse or rape described how the actions of care providers triggered distressing memories:

…my cervix was manually dilated forcefully after pleading for the Dr. to stop. This caused me to re-experience a previous rape. Later in my birth my Dr. performed a deep episiotomy after being told repeatedly that I did not want one… Images and fears from my past sexual abuse/assaults became constant in my mind after birth. (057)

…the whole experience was made worse as it triggered my post traumatic stress that related to gang rape in my teens. (444)

One woman felt that her birth experience was more traumatising than her experience of sexual abuse as a child:

…The most terrifying part of whole ordeal was being held down by 4 people and my genitals being touched and probed repeatedly without permission and no say in the matter, this is called rape, except when you are giving birth. My daughter’s birth was more sexually traumatising than the childhood abuse I’d experienced… (201)

## Discussion

This study described women’s experiences of birth trauma. The data set was large, and women recounted similar experiences across different birth settings and cultural contexts. The findings contribute to an understanding of birth trauma from the perspective of women experiencing it. Whilst non-interpersonal factors contributed to trauma, the majority of descriptions involved care provider actions and interactions. These findings are consistent with other studies that identify the relationship between the care provider and the woman as critical to the birth experience [20, 21, 37]. Whilst care providers may consider their actions and interactions to be routine, some woman experience them as traumatic [19]. Therefore, it is vital that care providers understand how their practice influences the psychological and emotional experience of birth, in addition to the physical outcome of birth.

In this study women described how care providers priorised their own agendas over the needs of the woman. This approach to practice is contrary to global standards regarding woman-centred maternity services [26, 38]. In addition, women felt that this resulted in unnecessary interventions, as care providers attempted to alter the birth process to fit their agenda. There is global concern regarding the increase in unnecessary medical intervention during birth [39, 40]. Therefore, this phenomena needs to be further examined as a possible contributing factor. In some cases, women in the study described how hospital staff observed or practiced on them to facilitate their learning. Whilst clinical learning is an important element of professional development, further research is needed to examine women’s experience of participation in these activities.

Women reported that their embodied knowledge about labour onset, progress, and fetal wellbeing was disregarded in favour of their care provider’s clinical evaluation. The clinical diagnosis of labour onset usually involves the assessment of contraction pattern and cervical dilatation [41]. However, this evaluation can conflict with women’s own perceptions regarding the onset of their labour [42, 43], causing distress [44–48]. Contradictory perceptions of progress can also occur during the expulsive phase of labour when women experience an uncontrollable urge to push [49]. Being instructed to resist the urge to push can be distressing for women [50, 51]. In this study, instructions to stop pushing were based on assumptions regarding normal labour timeframes, and on vaginal examinations. However, there is increasing debate in the literature regarding the accuracy of prescribed timeframes [52]; the efficacy of vaginal examinations [53]; and how clinical assessments relate to women’s experience of birth [49, 54, 55]. Whilst further research is necessary to examine women’s embodied knowledge of fetal wellbeing during labour, dismissal of women’s concerns has been found to contribute to the experience of trauma [56].

Consent is an important legal and ethical principle in health care [57]. For consent to be valid it must be voluntarily and feely given; the person consenting must not be under any undue influence or coercion; and there must be no misrepresentation as to the nature or necessity of the procedure. However, women in the study described being lied to, and threatened in order to gain their agreement for procedures. In particular, lies and threats centred on the wellbeing of the baby, and some women referred to this as ‘the dead baby threat’. Bohren et al. [56] also found that care providers threatened women regarding the safety of their baby in order to ensure they complied during labour. In addition, women in this study were threatened with being reported to child safety services if they did not agree to proposed procedures. Other studies have identified that women choosing birth options outside of the norm, such as freebirth, or homebirth after a caesarean, can experience threats relating to the safety of their baby, and of being reported to child safety agencies [13, 58].

Women’s accounts of birth trauma often included violence and physical abuse. Unfortunately these findings are not unique, and the World Health Organization [38] reports that many women worldwide experience disrespectful, abusive or neglectful treatment within maternity services. This phenomena has resulted in the introduction of the legal term ‘obstetric violence’ in some countries [59]. Women in the study used language associated with sexual assault and rape. Beck [19] also found that women likened the actions of care providers to rape; and Elmir et al. [21] noted that women used the term ‘birth rape’ to describe experiences of obstetric violence. Kitzinger [60] suggests that women who experience a traumatic birth display similar symptoms to rape survivors. In addition, women who have a history of sexual abuse or rape can have memories triggered by their care provider’s actions and interactions [19]. Montgomery et al. [61] carried out a study exploring the experience of birth for women with a history of childhood sexual abuse. They found it was not the intimate procedures themselves that triggered abuse memories. Instead, it was the manner in which the procedures were carried out. Actions and words that reduced a woman’s sense of control, and disempowered her could result in a ‘re-enactment of abuse’. These findings are consistent with this study, whereby descriptions of trauma focused on the manner in which actions were carried out, rather than on the physical procedures themselves.

A systematic review concluded that whilst the mistreatment of women in labour occurs at the level of care provider interactions, it is influenced by systematic failures at the health facility and health system level [56]. Current health systems are underpinned by a technocratic, biomedical paradigm in which the patient is considered passive, and authority and responsibility are inherent in the practitioner [62]. The power dynamics operating within this paradigm contribute to legitimising the control that care providers have over women, and subsequently to mistreatment [56]. Risk aversion and the avoidance of litigation is also a key component that influences care provider’s practice within the current technocratic maternity system [63–65]. However, concerns about litigation focus on perceived risks to physical outcomes for mothers and babies, rather than on psychosocial impacts of care [63–65].

Wagner [66] argues that dehumanising practices are so pervasive within maternity services, that care providers are unable to perceive them. He uses the analogy of fish being unable to see the water they swim in, to describe this phenomenon. This notion is supported by Bohern et al.’s [56] review that found some care providers consider the mistreatment of women to be normal. However, some care providers are cognisant of the paradigm in which they operate. In particular, research has demonstrated that midwives are often aware of an inherent conflict between woman-centred care, and the needs of the technocratic maternity system [67–69]. Midwives consciously adjust their practice to meet the cultural needs of the facilities in which they work in order to protect themselves professionally [67–69]. However, this results in what Hunter calls ‘emotional work’, as midwives practice in ways that are contradictory to their own woman-centred philosophy [68]. In addition, a recent study [70] found that midwives who witness interpersonal birth trauma can experience trauma themselves. The researchers suggest that witnessing this type of trauma may be perceived as a threat to their sense of personal and professional integrity.

Addressing interpersonal related birth trauma will require a multifold response on both a macro and micro level. Davis-Floyd [62] suggests that more effective woman-centred care can be delivered by combining humanism and holism with the current technocratic approach. However, this will require a cultural paradigm shift to support the evolution of such an approach. The World Health Organization recommends that five key actions should be taken to develop and sustain respectful maternity care for all women [38]. Firstly, greater support from governments and development partners for research and action on disrespect and abuse. Secondly, initiation and support of programs designed to improve the quality of maternal health care, with a strong focus on respectful care as an essential component of quality care. Thirdly, emphasising the rights of women to dignified, respectful care throughout their childbearing experience. Fourthly, the generation of data relating to respectful and disrespectful care practices, systems of accountability and meaningful professional support. Finally, the involvement of all stakeholders, including women, in efforts to improve quality care and eliminate disrespectful and abusive practices. In addition, it can be argued that the current risk discourse needs to be expanded to include psychosocial risk in addition to physical risk. On a micro level, Fenech and Thomson [7] suggest that care providers require training to develop their ability to prevent and identify trauma, and to respond sensitively to women’s emotional concerns.

### Limitations

The study was a cross-sectional qualitative study, therefore cannot establish cause and effect. There was a lack of representation across many countries with participants mainly from Australia and Oceania, North America, and Europe, and findings cannot be generalised globally. In addition, the data consisted of short written descriptions within a larger quantitative survey. In-depth qualitative accounts elicited by participant interviews may have enriched theme development.

## Conclusion

In this study women’s descriptions of childbirth trauma centred on the actions and interactions of care providers. Women described how care providers prioritised their own agendas; disregarded embodied knowledge; used lies and threats to gain compliance; and violated them. Findings contribute to the growing body of literature relating to women’s experiences of traumatic birth. Interpersonal birth trauma is becoming increasingly recognised as a global issue, and measures are required to address it. Recommendations include changing the current technocratic paradigm by including holistic and humanistic approaches to care delivery. Maternity service provision needs to be underpinned by the World Health Organization’s ‘five actions’ [38] to develop, promote and sustain respectful woman-centred care. Care providers require training and support to understand, value, and practice in ways that optimise psychological outcomes for women.

## Abbreviations

PTSD: Post traumatic stress disorder
